# Supplementary material for: Replication stress induces mitotic death through parallel pathways regulated by WAPL and telomere deprotection
Source: Nat Commun. 2019 Sep 17;10:4224. doi: 10.1038/s41467-019-12255-w (PMC6748914; doi:10.1038/s41467-019-12255-w)
Supplement: Supplementary file 1 — Supplementary Information [file 41467_2019_12255_MOESM1_ESM.pdf]

SUPPLEMENTARY INFORMATION

Replication stress induces mitotic death through parallel pathways regulated by WAPL and telomere deprotection

V. Pragathi Masamsetti, Ronnie Ren Jie Low, Ka Sin Mak, Aisling O'Connor, Chris D. Riffkin, Noa Lamm, Laure Crabbe, Jan Karlseder, David C.S. Huang, Makoto T Hayashi & Anthony J. Cesare

Supplementary Figures 1-8

Supplementary Movies 1-4

Supplementary Figure 1

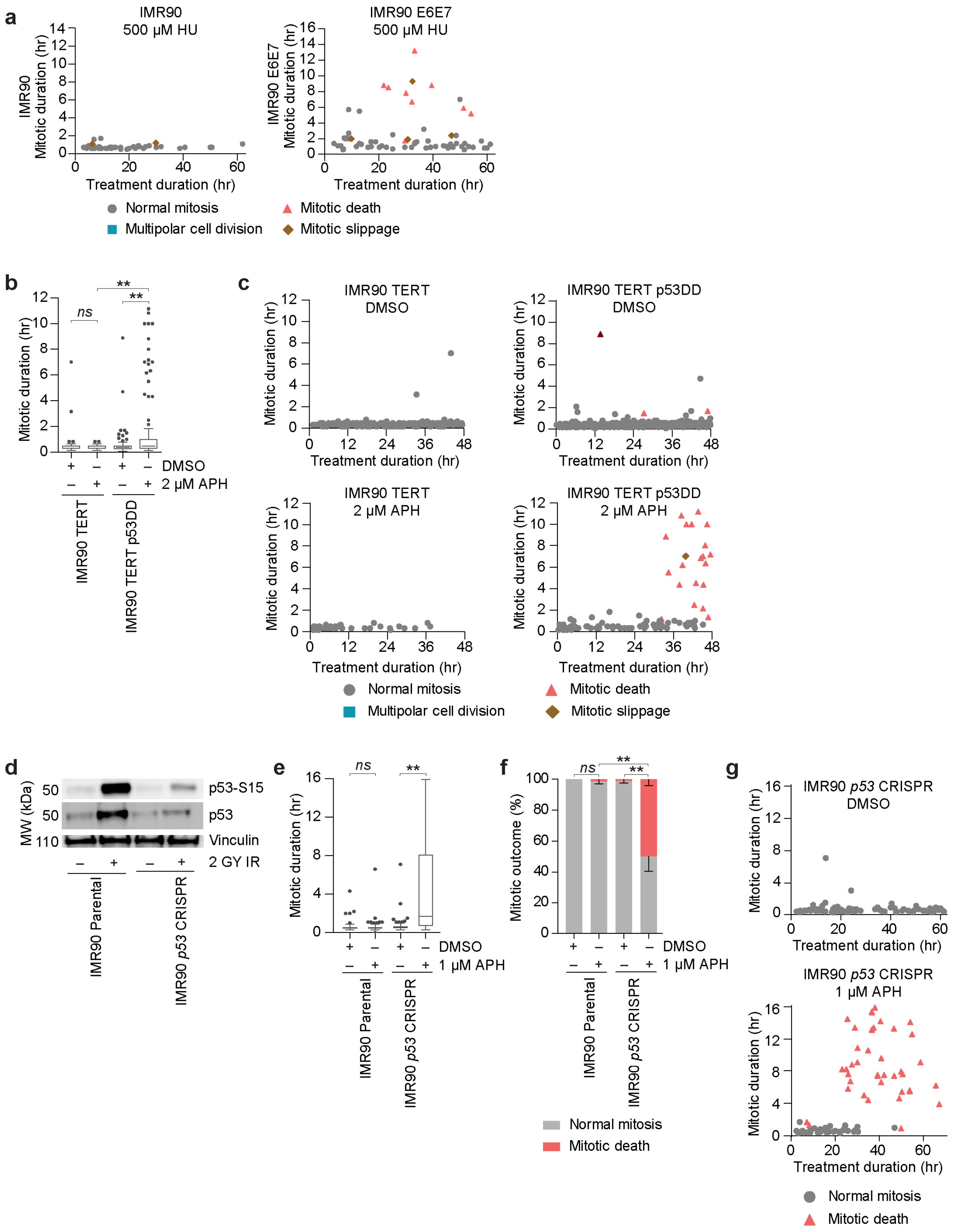

Supplementary Figure 1. Lethal replication stress induces mitotic lethality in p53 compromised IMR90 fibroblasts. a) Two-dimensional plots of mitotic duration and outcome from IMR90 and IMR90 E6E7 treated with Hydroxyurea (HU) (data corresponding to Fig. 1c, d). Data are presented as described in Fig. 1d (n = 46 mitoses for IMR90 and 66 mitoses for IMR90 E6E7 compiled from three biological replicates into a single graph). **b**) Mitotic duration of IMR90-TERT and IMR90-TERT p53DD treated with DMSO or Aphidicolin (APH) (Representative replicate showing ≥ 43 mitoses per condition compiled in a Tukey box plot, Mann-Whitney test). **c**) Two-dimensional plots of the data shown in (**b**). **d**) Western blots of whole cell extracts from IMR90 parental and IMR90 *p53* CRISPR cell cultures. Cultures treated with two gray of ionizing radiation (2 GY IR) are include as a positive control of p53 pathway activation. **e**) Mitotic duration of IMR90 parental and *p53* CRISPR cultures following treatment with DMSO or APH (n = 2 biological replicates scoring n ≥ 85 mitotic events per condition compiled in a Tukey box plot, Mann-Whitney test). **f**) Outcome of the mitotic events in (**e**) (mean ± s.e.m, n = 2 biological replicates, Fisher's Exact Test). **g**) Two-dimensional dot plots of mitotic duration and outcome from (**e**, **f**). Source data are provided as a Source Data file.

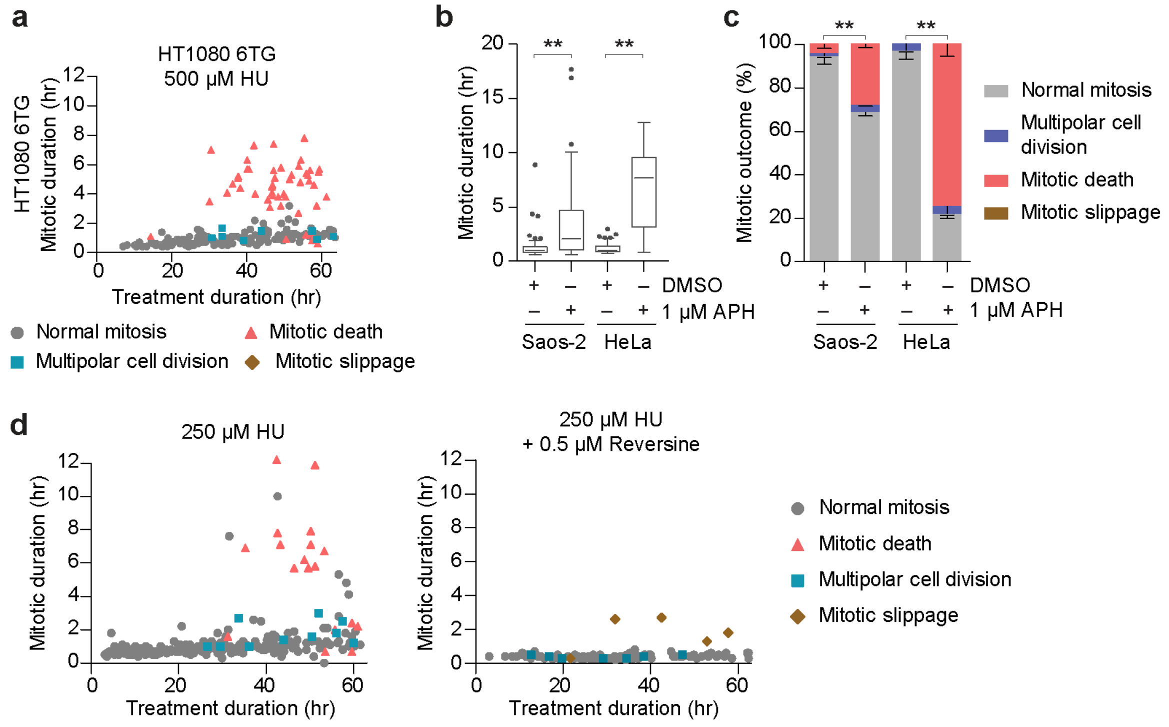

Supplementary Figure 2. Lethal replication stress induces mitotic lethality in cancer cell lines. a) Two-dimensional plot of HT1080 6TG cells treated with 500 μM HU (data corresponding to Fig. 1g, h; n ≥ 200 mitoses compiled from three biological replicates into a single graph). **b**) Mitotic duration of Saos-2 and HeLa cells treated with DMSO or APH (three biological replicates scoring ≥ 43 mitoses per condition compiled in a Tukey box plot, Mann-Whitney test, *p < 0.01). **c**) Outcome of the mitotic events in (**b**) (mean ± s.e.m, n = 3 biological replicates, Fisher's Exact Test). **d**), Two-dimensional plots of HT1080 6TG cells treated with reversine ± HU (data corresponding to Fig. 1j, k; three biological replicates scoring n ≥ 112 mitoses per condition compiled into a single graph). Source data are provided as a Source Data file.

Supplementary Figure 3

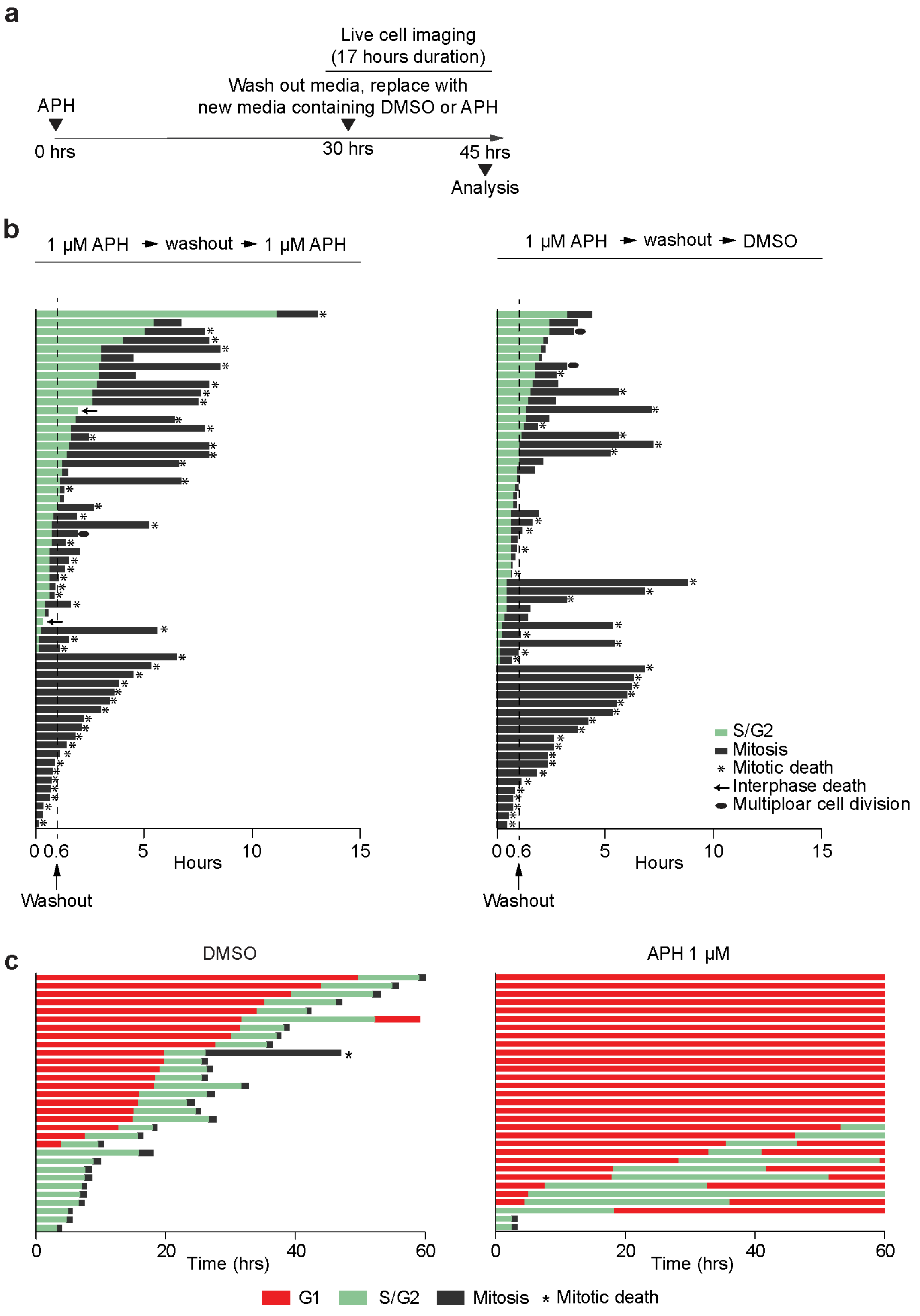

Supplementary Figure 3: Mitotic arrest and death in p53-compromised cells occurs independent of mitotic APH treatment, and replication stress in p53-competent fibroblasts induces mitotic bypass. a) Timeline of experiments in (**b**). **b**) Cell fate map of APH washout experiment in HT1080 6TG FUCCI cells. Cells were treated with APH for 30 hours before media washout and replacement with fresh media containing APH or DMSO. The dotted line shows when the media was washed out and replaced. Each bar represents an individual cell as it progresses through cell division or to death. Segment length represents the duration the cell spent in each cell cycle phase, which are color coded according to the legend. Data are from 2 independent biological replicates compiled into a single graph (n = 58 mitoses for each condition). **c**) Fate map of IMR90-FUCCI cells treated with DMSO or APH. Each bar represents an individual cell as it progresses through the first cell division, mitotic bypass, or cell death, relative to DMSO or APH treatment (T=0). Segment length represents the duration a cell spent in that cell cycle phase. One of three independent biological replicates are shown (n = 31 cells for each condition). Source data are provided as a Source Data file.

Supplementary Figure 4

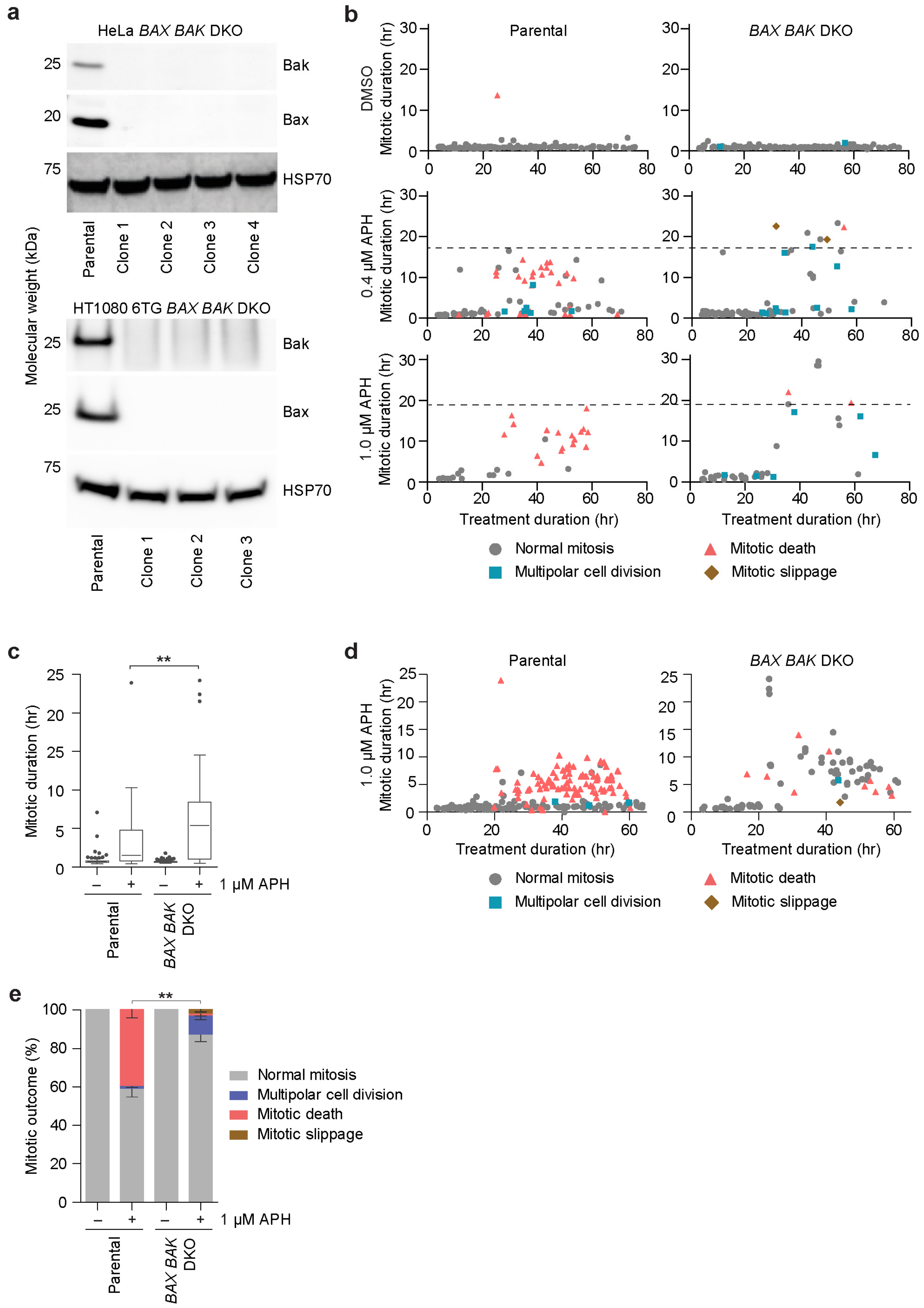

Supplementary Figure 4: Replication stress-induced mitotic death is primarily BAX/BAK-dependent. a) Western blots of whole cell extracts from HeLa (upper panels) and HT1080 6TG (lower panels) parental and *BAX BAK* DKO clones. **b**) Two-dimensional graphs of mitotic duration and outcome from HeLa parental and *BAX BAK* DKO cells shown in Fig 3a, b ($n \geq 32$ mitoses compiled from three biological replicates of independent DKO clones compiled into a single graph). The dashed line represents the longest mitotic event observed in the corresponding parental cells treated with the indicated dose of APH. **c**) Mitotic duration of HT1080 6TG parental and *BAX BAK* DKO cells treated with DMSO or APH (three biological replicates on independent DKO clones scoring ≥ 91 mitoses per condition are compiled into a Tukey box plot, Mann-Whitney test). **d**) Two-dimensional representation of mitotic duration and outcome of the data in (**c** and **e**). **e**) Mitotic outcome of the cells from (**c** and **d**, mean ± s.e.m, $n = 3$ biological replicates Fisher's exact test). For all panels ** $p < 0.01$. Source data are provided as a Source Data file.

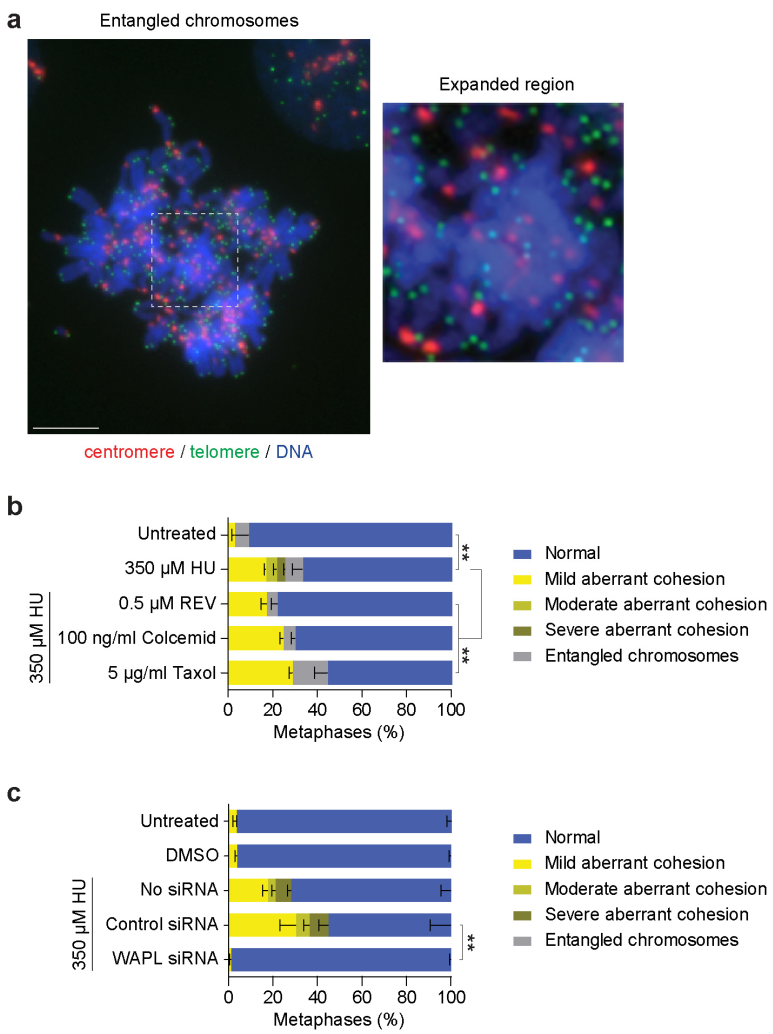

Supplementary Figure 5: Lethal replication stress-induces entangled chromosomes and cohesion fatigue. a) Example image of entangled chromosomes from HT1080 6TG cells, stained with DAPI (blue), and PNA FISH against the centromeres (red) and telomeres (green). Scale bar represents 10 µm. **b**) Quantitation of cohesion phenotypes as depicted in Fig. 4b in HT1080 6TG cells treated with DMSO or HU ± reversine (REV), colcemid or Taxol (mean ± s.e.m, n = 3 biological replicates scoring ≥ 70 chromosome spreads per condition, Fisher's Exact Test.). **c**) Quantitation of cohesion phenotypes depicted in Fig. 4b in HT1080 6TG cells treated with HU ± siRNA (mean ± s.e.m, n= 3 biological replicates scoring ≥ 180 chromosome spreads per condition, Fisher's Exact Test). For all panels, **p < 0.01. Source data are provided as a Source Data file.

Supplementary Figure 6

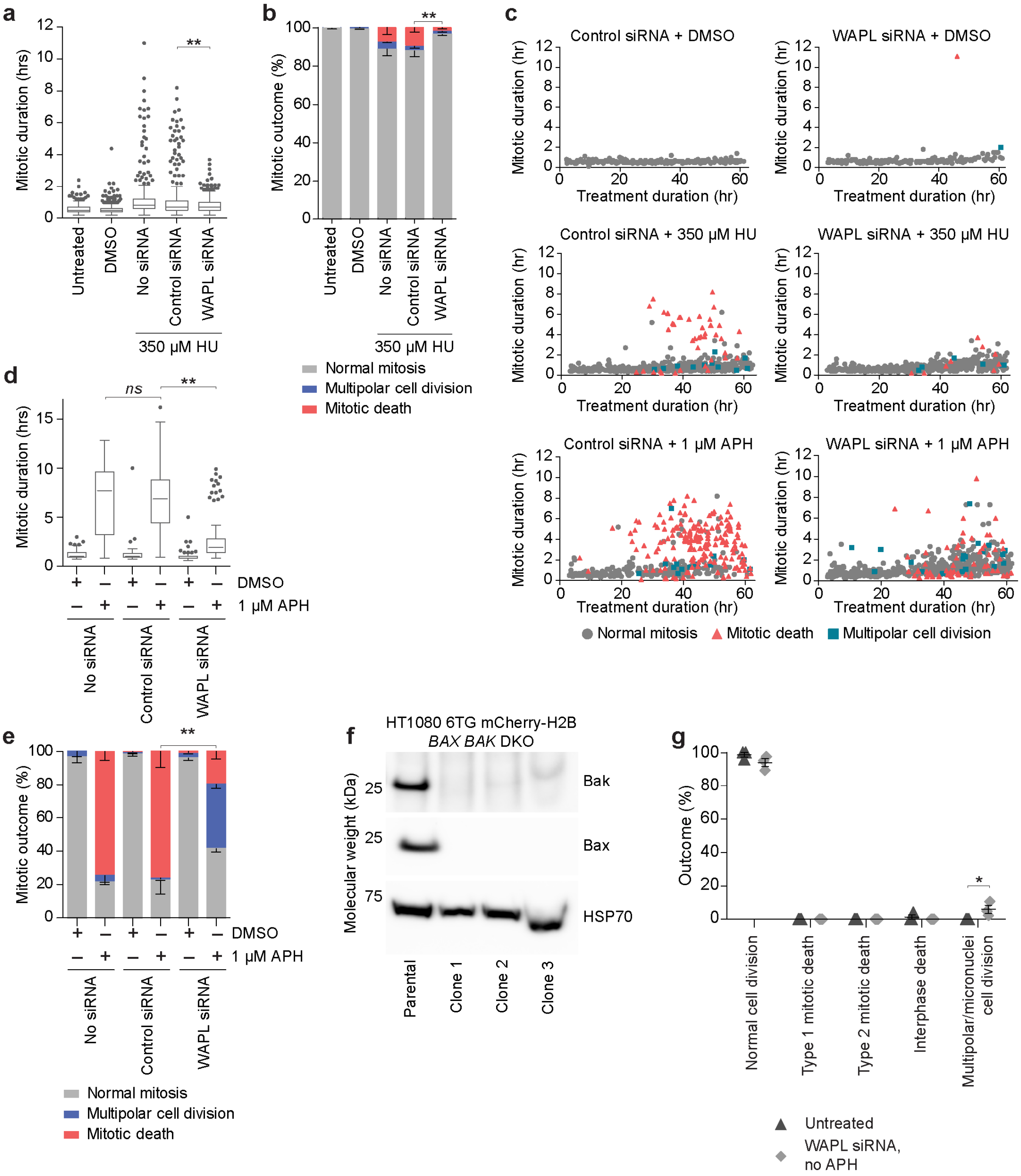

Supplementary Figure 6: WAPL depletion rescues cohesion fatigue, mitotic arrest, and mitotic cell death in cells treated with lethal doses of APH or HU. a) Mitotic duration of HT1080 6TG cells treated with HU ± siRNA (three biological replicates scoring ≥ 413 mitoses per condition are compiled into a Tukey box plot, Mann-Whitney test). **b**) Mitotic outcome of the cells in (**a**) (mean ± s.e.m., n = 3 biological replicates, Fisher's Exact Test). **c**) Two-dimensional representation of mitotic duration and outcome of the data in Fig. 5a, b and (**a**, **b**) above. **d**) Mitotic duration of HeLa cells treated with DMSO and APH ± control or WAPL siRNA (three biological replicates scoring ≥ 43 mitoses per replicate are compiled into a Tukey box plot, Mann-Whitney test). **e**) Mitotic outcome of the cells in (**e**) (mean ± s.e.m., n = 3 biological replicates, Fisher's Exact Test). **f**) Western blot of HT1080 6TG-H2B mCherry parental and *BAX BAK* DKO clones. **g**) Mitotic outcome of WAPL depleted HT1080 6TG H2B-mCherry cells in the absence of APH (mean ± s.e.m., n = 3 biological replicates biological scoring ≥ 111 mitoses per condition). For all panels, *ns* = not significant, $*p < 0.05$, $**p < 0.01$. Source data are provided as a Source Data file.

Supplementary Figure 7

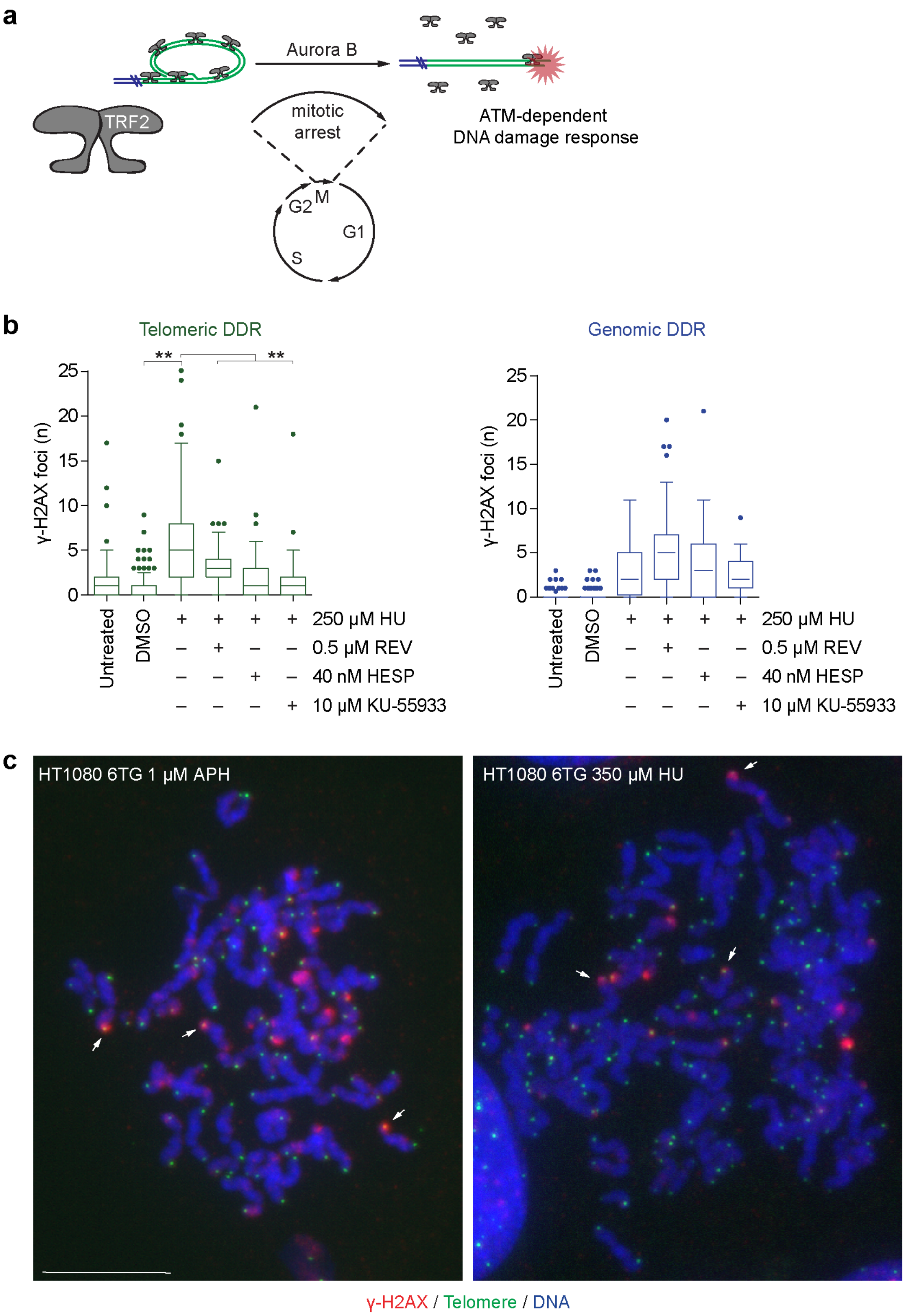

Supplementary Figure 7: The telomere DDR induced by lethal replication stress is dependent on mitotic arrest, Aurora B, and ATM. a) Graphical depiction of non-canonical, mitotic arrest-dependent telomere deprotection[1,2]. **b**) Quantitation of mitotic telomeric and genomic DDR foci in HT1080 6TG cells treated with HU ± REV, Hesperidin (HESP), or KU-55933 (three biological replicates scoring n ≥ 37 chromosome spreads per replicate are compiled in a Tukey box plot, Mann-Whitney test, $^{**}p < 0.01$). **c**) Representative image of cytocentrifuged chromosome spreads from APH or HU treated HT1080 6TG cells displaying telomere DDR foci and separated sister chromatids. Examples of telomere DDR foci on separated sister chromatids are indicated by the white arrows. Samples are stained with DAPI (blue), γ-H2AX immunofluorescence (red) and telomere FISH (green). Scale bar represents 10 μm. Source data are provided as a Source Data file.

Supplementary Figure 8

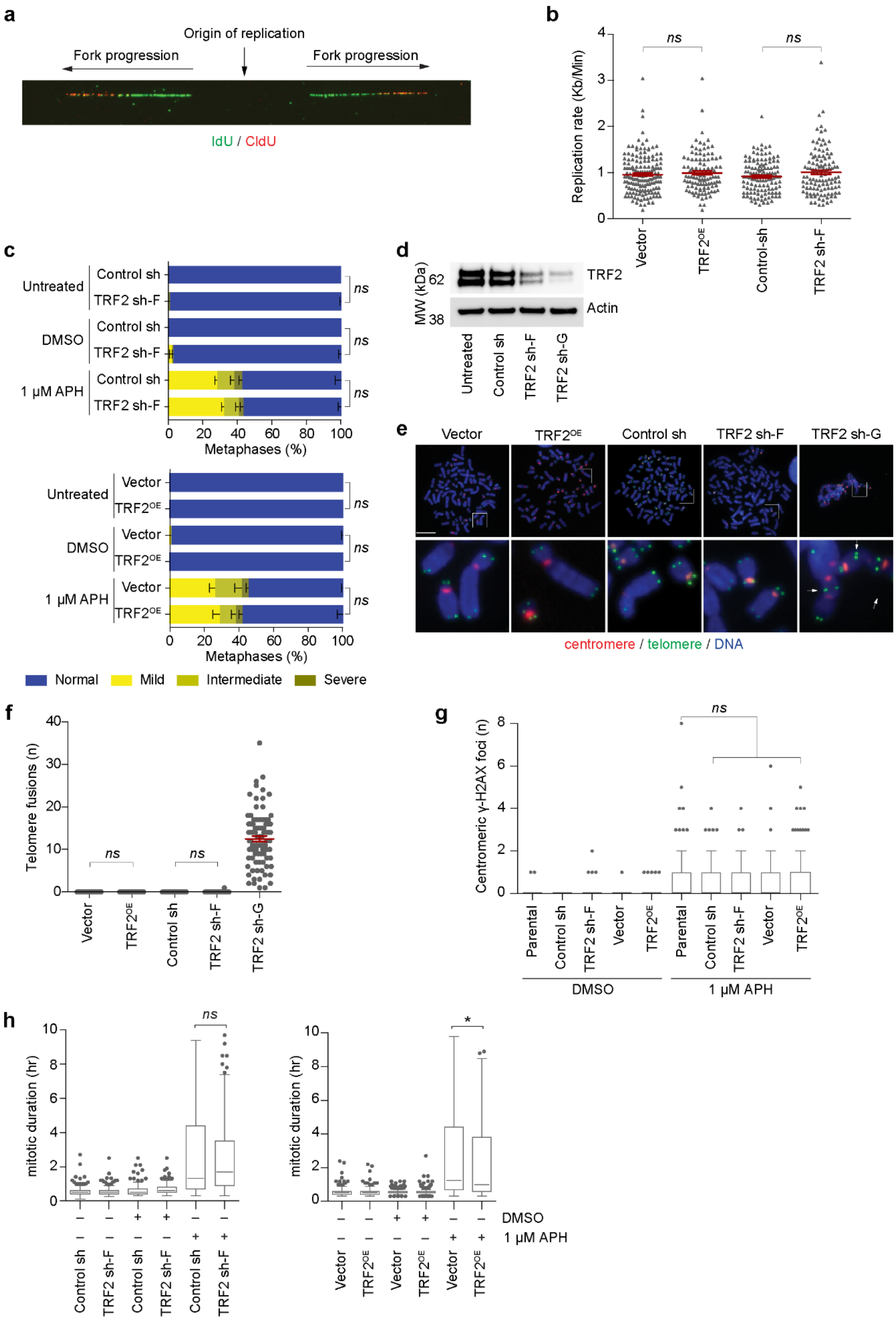

Supplementary Figure 8: TRF2 sh-F and TRF2^{OE} do not induce telomere fusions, nor impact replication rates, cohesion status or centromere γ-H2AX foci. a) Representative image of DNA fiber analysis to measure replication rates. **b**) Genomic DNA Replication rates in HT1080 6TG control, TRF2 sh-F, and TRF2^{OE} (all replication forks from two biological replicates are compiled into a dot plot, n ≥ *109* total forks measured per condition, mean ± s.e.m., Student's t-test). **c**) Quantitation of cohesion phenotypes as shown in Fig. 4b in HT1080 6TG cells transduced with the indicated vectors, treated with DMSO or APH (mean ± s.e.m of n = 3 experiments scoring ≥ 50 chromosome spread per replicate, Fisher's Exact Test). **d**) Western blots of whole cell extracts from HT1080 6TG cells transduced with TRF2 sh-F and the more effective TRF2 sh-G[36]. **e**) Representative cytogenetic chromosome spreads from HT1080 6TG cells transduced with the indicated vectors. Telomere-telomere fusions are indicted by arrows in TRF2 sh-G. Scale bar represents 10 µm. **f**) Quantitation of telomere-telomere fusions in mitoses from the conditions in (**e**) (three biological replicates scoring ≥ 30 mitotic spreads per replicate are compiled into a dot plot, mean ± s.e.m., Mann-Whitney test). **g**) Quantitation of mitotic centromere γ-H2AX foci from Control sh, TRF2 sh-F, vector, and TRF2^{OE} cells HT1080 6TG cells ± DMSO or APH (three biological replicates scoring n = 50 mitotic spreads per replicate compiled in a Tukey box plot, one-way ANOVA). **h**) Mitotic duration of HT1080 6TG control, TRF2 sh-F, and TRF2^{OE} cells treated with DMSO or APH (three biological replicates scoring n ≥ 267 mitoses per condition are compiled in a Tukey box plot, Mann-Whitney test). For all panels, *ns* = not significant, * $p < 0.05$. Source data are provided as a Source Data file.

Supplementary References

1. Hayashi, M.T., Cesare, A.J., Fitzpatrick, J.A., Lazzerini-Denchi, E. & Karlseder, J. A telomere-dependent DNA damage checkpoint induced by prolonged mitotic arrest. Nat Struct Mol Biol 19, 387-94 (2012).

2. Van Ly, D. et al. Telomere Loop Dynamics in Chromosome End Protection. Mol Cell 71, 510-525 (2018).
