# Supplementary material for: Replication stress induces mitotic death through parallel pathways regulated by WAPL and telomere deprotection
Source: Nat Commun. 2019 Sep 17;10:4224. doi: 10.1038/s41467-019-12255-w (PMC6748914; doi:10.1038/s41467-019-12255-w)
Supplement: Supplementary file 3 — Description of Additional Supplementary Files [file 41467_2019_12255_MOESM3_ESM.pdf]

Description of Additional Supplementary Files

File Name: Supplementary Movie 1
Description: **Lethal replication stress induces mitotic death.** Live cell imaging of HT1080 6TG cells treated with DMSO or 1 µM APH. Imaging duration is shown in the top left corner as (day:hr). Some examples of mitotic cell death events are indicated with a red > symbol.

File Name: Supplementary Movie 2
Description: **Lethal replication stress induces mitotic death in the immediately following mitosis.** Live cell imaging of HT1080 6TG FUCCI cells (Sakaue-Sawano et al. 2008) treated with DMSO or APH at t=0 hours. G1 phase cells are indicated by mCherry-hCdt1(30/120) stability and S/G2 phase cells by mVenus-hGeminin (1/110) stability. Duration of live cell imaging was shown on the top left corner of the movie (day:hr). Some examples of mitotic cell death events are indicated with a yellow >.

File Name: Supplementary Movie 3
Description: **Two types of mitotic cell death are induced by replication stress.** Spinning disk confocal live cell imaging of HT1080 6TG H2B-mCherry cells treated with 1 µM APH. Brightfield image was captured with differential interference contrast. Examples of Type 1 and Type 2 mitotic death are indicated. Time is shown as minutes.

File Name: Supplementary Movie 4
Description: **Mitotic death escape in BAX BAK DKO cells induces multilobular nuclei.** Spinning disk confocal live cell imaging of HT1080 6TG H2B-mCherry BAX BAK DKO cells treated with 1 µM APH. Time is shown as hours.
