# Supplementary material for: Replication stress induces mitotic death through parallel pathways regulated by WAPL and telomere deprotection
Source: Nat Commun. 2019 Sep 17;10:4224. doi: 10.1038/s41467-019-12255-w (PMC6748914; doi:10.1038/s41467-019-12255-w)
Supplement: Supplementary file 8 — Reporting Summary [file 41467_2019_12255_MOESM8_ESM.pdf]

nature research

Corresponding author(s): Cesare, Anthony J.

Last updated by author(s): Jul 25, 2019

Reporting Summary

Nature Research wishes to improve the reproducibility of the work that we publish. This form provides structure for consistency and transparency in reporting. For further information on Nature Research policies, see Authors & Referees and the Editorial Policy Checklist.

Statistics

For all statistical analyses, confirm that the following items are present in the figure legend, table legend, main text, or Methods section.

n/a	Confirmed	
☐	☒	The exact sample size (n) for each experimental group/condition, given as a discrete number and unit of measurement
☐	☒	A statement on whether measurements were taken from distinct samples or whether the same sample was measured repeatedly
☐	☒	The statistical test(s) used AND whether they are one- or two-sided<br>*Only common tests should be described solely by name; describe more complex techniques in the Methods section.*
☒	☐	A description of all covariates tested
☒	☐	A description of any assumptions or corrections, such as tests of normality and adjustment for multiple comparisons
☐	☒	A full description of the statistical parameters including central tendency (e.g. means) or other basic estimates (e.g. regression coefficient) AND variation (e.g. standard deviation) or associated estimates of uncertainty (e.g. confidence intervals)
☐	☒	For null hypothesis testing, the test statistic (e.g. F, t, r) with confidence intervals, effect sizes, degrees of freedom and P value noted<br>*Give P values as exact values whenever suitable.*
☒	☐	For Bayesian analysis, information on the choice of priors and Markov chain Monte Carlo settings
☒	☐	For hierarchical and complex designs, identification of the appropriate level for tests and full reporting of outcomes
☒	☐	Estimates of effect sizes (e.g. Cohen's d, Pearson's r), indicating how they were calculated

Our web collection on statistics for biologists contains articles on many of the points above.

Software and code

Policy information about availability of computer code

Data collection	Software packages used to collect data: ZEISS Zen, Metasystems, Fuji film Multigauge (Western blot visualization)
Data analysis	Software packages used to analyze data: Imaris, GraphPad Prism, Metasystems, Excel.

For manuscripts utilizing custom algorithms or software that are central to the research but not yet described in published literature, software must be made available to editors/reviewers. We strongly encourage code deposition in a community repository (e.g. GitHub). See the Nature Research guidelines for submitting code & software for further information.

Data

Policy information about availability of data

All manuscripts must include a data availability statement. This statement should provide the following information, where applicable:

- Accession codes, unique identifiers, or web links for publicly available datasets
- A list of figures that have associated raw data
- A description of any restrictions on data availability

The data that support the findings of this study are archived at the Children's Medical Research Institute, Kyoto University, or the Walter and Eliza Hall Institute of Medical Research, and are available from the corresponding author upon reasonable request. The source data underlying all the presented western blots, histograms, and dot plots are available in the Source Data file.

Field-specific reporting

Please select the one below that is the best fit for your research. If you are not sure, read the appropriate sections before making your selection.

☒ Life sciences ☐ Behavioural & social sciences ☐ Ecological, evolutionary & environmental sciences

For a reference copy of the document with all sections, see nature.com/documents/nr-reporting-summary-flat.pdf

Life sciences study design

All studies must disclose on these points even when the disclosure is negative.

Sample size	We did not perform statistical analysis to predetermine sample sizes. However, our sample sizes were similar or in excess of those published elsewhere and are described in the text.
Data exclusions	No data were excluded
Replication	All experiments were repeated multiple times, typically three or four independent experiments. Whenever possible experiments were also completed in multiple cell lines.
Randomization	Areas to be imaged using live-cell microscopy were selected at random within the culture. Upon completion of imaging, cells were analyzed throughout the duration of the movies. Images of fixed samples were collected using automated microscopy. This resulted in a series of images. One of these images was selected at random and all cells from that image, and the following sequential images, were analyzed until data from a predetermined number of cells were attained.
Blinding	Investigators were not blinded.

Reporting for specific materials, systems and methods

We require information from authors about some types of materials, experimental systems and methods used in many studies. Here, indicate whether each material, system or method listed is relevant to your study. If you are not sure if a list item applies to your research, read the appropriate section before selecting a response.

Materials & experimental systems

n/a	Involved in the study	
☐	☒	Antibodies
☐	☒	Eukaryotic cell lines
☒	☐	Palaeontology
☒	☐	Animals and other organisms
☒	☐	Human research participants
☒	☐	Clinical data

Methods

n/a	Involved in the study	
☒	☐	ChIP-seq
☒	☐	Flow cytometry
☒	☐	MRI-based neuroimaging

Antibodies

Antibodies used	A list of antibodies is included in the methods section under the subheading "Antibodies".
Validation	We used well validated antibodies from commercial sources. Where applicable, antibodies were validated by siRNA depletion or through control experiments to induce DNA damage response activation.

Eukaryotic cell lines

Policy information about cell lines

Cell line source(s)	The source of each cell line was indicated in the methods section under "Cell culture and treatments".
Authentication	All cell lines were validated by Cell Bank Australia using short tandem repeat profiling as indicated in the methods in the "Cell Culture and treatments" section.
Mycoplasma contamination	All cell lines were identified as mycoplasma negative as indicated in the "Cell culture and treatments" portion of the methods section.
Commonly misidentified lines (See ICLAC register)	No commonly misidentified cell lines were used in this study.
